# Improvement by Medication Less than Expected in Parkinson's Disease: Blinded Evaluation of Levodopa Response

**DOI:** 10.1155/2024/2649578

**Published:** 2024-02-21

**Authors:** Mette Niemann Johansen, Anna Handberg, Mohamed El Haddouchi, Josefine Grundtvig, Steen Rusborg Jensen, Lisette Salvesen, Annemette Løkkegaard

**Affiliations:** ^1^Department of Neurology, Bispebjerg Hospital, Copenhagen, Denmark; ^2^Emergency Department, Zealand University Hospital Køge, Køge, Denmark; ^3^Department of Otorhinolaryngology, Head and Neck Surgery & Audiology, Copenhagen University Hospital, Copenhagen, Denmark; ^4^Department of Clinical Medicine, Copenhagen University, Copenhagen, Denmark

## Abstract

**Background:**

The latest Movement Disorder Society (MDS) diagnostic criteria require a good and sustained response to medication to get a diagnosis of Parkinson's disease, PD.

**Objective:**

The aim of this study was to evaluate levodopa response in a group of patients with probable PD, diagnosed by movement disorder specialists.

**Methods:**

An acute levodopa challenge test (LDCT) was performed after pausing the dopaminergic medication for 6 half-times. The motor part of the Unified Parkinson's Disease Rating Scale was performed in the OFF-state and after LDCT (ON). A good effect was defined as >30% improvement. A video-protocol was used to secure standardized motor examination with blinded assessments of the UPDRS-III OFF and ON. An age-matched group of control subjects (CS) was included but did not go through LDCT. All participants were evaluated with Montreal Cognitive Assessment (MoCA) and Beck's Depression Inventory (BDI).

**Results:**

In the statistical analysis, 37 patients were included. Twenty-one patients showed an improvement ≤30%, while 16 patients showed an improvement >30%. LDCT showed an overall mean improvement of 27.3% of motor UPDRS. In 43.2%, there was a discrepancy between the effect seen with the LDCT and the patients' self-perceived medicine evaluation. Patients with PD had a significantly lower MoCA score and more depressive symptoms compared to CS.

**Conclusions:**

We showed an acute effect of levodopa using LDCT that was around 30% improvement. While it lends support to the use of this limit in the MDS diagnostic criteria, an acute effect of less than 30% should be considered acceptable in some patients. Our study highlights a discrepancy in the objective measure of medicine effect on motor symptoms and the patient's subjective evaluation.

## 1. Introduction

Parkinson's disease (PD) is the second most common neurodegenerative disease, causing severe disability with increasing prevalence. A diagnosis of PD is based on the motor manifestations: bradykinesia, rigidity, and tremor, and clinical symptoms related to the loss of dopamine. In PD, these symptoms of parkinsonism are related to a neurodegenerative pathological process involving misfolding and aggregation of *α*-synuclein and formation of inclusion bodies, Lewy bodies, primarily in dopaminergic neurons. A diagnosis of PD is clinicopathological and requires both this pathological hallmark as well as the clinical phenomenology. In 2015, the Movement Disorder Society (MDS) put forward updated clinical diagnostic criteria for PD to increase early diagnostic accuracy in patients with Lewy body pathology Parkinson's disease [1]. Dopaminergic neuronal loss in addition to Lewy body formation is central in the pathology of PD. In the diagnostic criteria from 2015, the importance of this pathological hallmark is emphasized even more than in earlier diagnostic criteria [2], through suggestion of operational criteria including (i) support by objective measurement of effect on motor symptoms showing a >30% improvement with dopaminergic medication in the motor part of the Unified Parkinson's Disease Rating Scale (UPDRS-III), (ii) a clearly documented history of marked changes with medication from the patient or the caregiver, and also that (iii) lack of sufficient motor improvement should be an exclusion criterion [3].

While the diagnostic criteria of PD from MDS have been validated against expert clinical diagnosis and the United Kingdom Brain Bank criteria from 1988 [4], the clinical importance of having this definition of the effect of medication in a movement disorder clinic may still be more thoroughly evaluated.

Treatment with levodopa (L-dopa) is the most efficient dopaminergic therapy and the golden standard treatment for PD [1]. An evaluation of dopaminergic effect is therefore necessarily based on treatment with L-dopa. To evaluate the treatment effect in an objective manner, the standardized acute levodopa challenge test (LDCT) can be used to assess motor symptoms both OFF and ON medication to evaluate the L-dopa response. Besides using LDCT in a diagnostic approach, the acute LDCT protocol has been described and used in the measurement of disease progression [5]. In addition, it has been widely accepted as an evaluation of levodopa effect as a preoperative evaluation before deep brain stimulation of the subthalamic nucleus (STN-DBS) [6].

While a dopaminergic response in patients with PD is the common finding, in our tertiary movement disorder clinic, we, from time to time, encounter patients who report a less than good response to medication but otherwise fulfill the clinical diagnostic criteria for probable PD and do not exhibit any other red flags for atypical parkinsonism.

The aim of this study was therefore to explore the validity of the current diagnostic criteria, regarding the requirement of improvement of motor symptoms with L-dopa and to assess the L-dopa response as a diagnostic tool in a group of patients fulfilling diagnostic criteria for probable PD.

To do so, patients were asked, whether they had a clearly documented history of marked changes with medication and if they believed, they were responding. In addition, the standardized acute levodopa challenge test (LDCT) was used to assess motor symptoms both OFF and ON medication to evaluate the L-dopa response objectively.

## 2. Materials and Methods

Patients diagnosed with probable PD by a movement disorder specialist were included from the movement disorders' clinic at the Department of Neurology at Bispebjerg Hospital, Denmark. Patients with both a self-reported good and poor response to medication were included. Inclusion criteria were at least 3 years of PD symptoms and an age ≥45 years. Control subjects (CS) were recruited among relatives and through research ads. The inclusion criteria for the CS were no neurological or psychiatric history or diseases and an age between 45 and 90 years. CS were matched for sex, age, and years of education. All participants in this prospective cohort were recruited form March 2019 to December 2021. Exclusion criteria were a clinical diagnosis of dementia, or the Montreal Cognitive Assessment (MoCA) score <19, or if the participants could not cooperate to the examination/tests. Furthermore, participants who took antidopaminergic medication were not included.

The acute LDCT is a well-defined and widely used drug challenge test which in this study is used to evaluate the L-dopa effect in the patients with PD. Before the LDCT, patients paused their antiparkinsonian medication for at least 6 half-times, i.e., for levodopa 12 hours and for dopamine agonists 72 hours, so that the evaluation of motor symptoms could be as precise as possible with regard to a comparison without medication (OFF ) and with medication (ON). The day of the LDCT, the patients were examined by the same investigator and always in the morning after >12 hours pause of levodopa overnight. The evaluation in the OFF-state contained an interview about the patients' medical history, and they were asked to self-rate their usual effect of medication. The patients were evaluated in the OFF-state with testing of motor symptoms using the Unified Parkinson's Disease Rating Scale, the motor part UPDRS-III [7]. The patients were all given 200/25 mg soluble levodopa/benserazid (Madopar Quick©), and one hour after drug administration, they were evaluated for the effect using UPDRS-III in the defined ON-state. Some patients had a marked effect earlier. A total UPDRS was performed (including UPDRS-I, mental and cognitive symptoms, UPDRS-II, ADL function, UPDRS-III, motor symptoms and UPDRS-IV, and side effects to medication), as well as a screening for cognitive changes with MoCA and depressive symptoms with Beck's Depression Inventory (BDI) [8]. The CS completed the MoCA and BDI, and instead of the UPDRS, a neurological examination was performed.

The protocol was changed after the first 9 patients to secure an unbiased evaluation. Hence, in the large majority of the patients, we used a video protocol to secure standardized motor examination with blinded assessments. The UPDRS-III was thus first rated by the investigator (MNJ) and afterwards videotaped. Later, the videotaped UPDRS-III OFF and ON were assessed blinded by the same two specialists (AL, SRJ).

### 2.1. Statistical Analysis

Nominal and ordinal variable expressed as median with minimum and maximum values and parameters were compared with the Mann–Whitney *U* test. Binary data were described as absolute frequency and relative frequency in percentage, and parameters were compared with Fisher's exact test. Motor improvement of UPDRS-III ON compared to OFF was graphically illustrated with a scatter plot.

The significance level was set at 5%. All analyses were performed with the statistic software R (version 4.1.0; R Foundation for Statistical Computing, Austria, Vienna).

The study was approved by the Danish National Committee on Health Research Ethics, VEK (H-18055648), and data protection agencies, and the study is registered on https://clinicaltrials.gov. All participants gave written informed consent prior to participation.

## 3. Results

A total of 38 patients with PD and 30 CS were included. One patient and one CS were excluded due to a MoCA score <19. Data from the 37 patients and 29 CS were included in the statistical analysis. Patients and CS were matched for age, gender, and education. The patients with PD had a median age of 66 years, and the CS had a median age of 63 years (*p*=0.522). 40.5% of patients with PD were female compared to 55.2% in the CS group (*p*=0.321). Demographic data are shown in Table 1.

**Figure 1:**
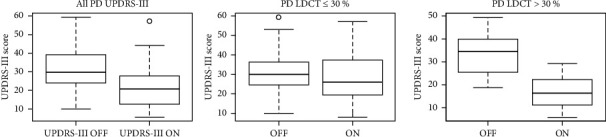
The first boxplot shows the UPDRS-III scores in both OFF-state and ON-state for all the patients with PD. The second boxplot shows the UPDRS-III OFF and ON scores for patients with ≤30% motor improvement in the acute LDCT, and the third boxplot shows the scores for patient with >30% motor improvement.

**Figure 2:**
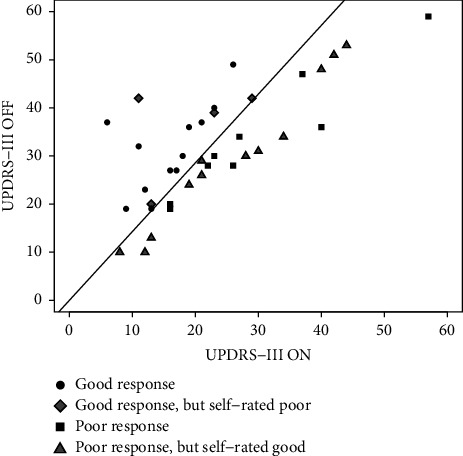
Motor improvement ON compared to OFF in UPDRS-III for all patients with PD. The oblique line shows cutoff 30%. 12 PD had self-rated a good medicine response, but we measured an L-dopa response ≤30%. On the other hand, 4 PD had self-rated a poor medicine response, but we measured a good L-dopa response.

The effect of levodopa in the LDCT was defined as more than a 30% improvement of UPDRS-III in the defined ON-state compared to the OFF-state. The UPDRS-III scores in both OFF and ON state for all participants with PD are shown in the first boxplot in Figure 1. The patients with PD were identified who had either an improvement of ≤30% or >30% in the acute LDCT. Twenty-one patients showed an improvement ≤30%, while 16 patients showed an improvement >30%. In Figure 1, the OFF and ON scores for each group are depicted. We found no difference in age, sex, disease duration, or MoCA score depending on LCDT (Table 2). The motor improvement in the LDCT (ON compared to OFF) for all patients with PD is graphically shown in Figure 2. According to the patient's previously self-reported effect of medication, 13 patients had described a poor L-dopa response and 24 patients a good L-dopa response. In 43.2%, there was a discrepancy between the subjective evaluation of the usual response to medicine compared to the effect objectively measured with the LDCT (Figure 2). The positive predictive value (PPV) of a good LCDT was 75%, while a PPV of a negative LCDT was 43%. Nine patients (24%) had both a subjective report of the poor treatment effect and a negative LCDT.

Motor function and test results in patients with a LCDT response of either over or under 30% are shown in Table 3. The UPDRS in the OFF-state did not differ between the groups (*p*=0.634). There were no significant differences in UPDRS-II (activities of daily living) or modified Hoehn and Yahr (H&Y). There was no significant difference in the total L-dopa equivalent daily dose (LEDD); patients with LCDT ≤ 30% had a mean total LEDD of 550 mg/day and patients with good response 612 mg/day (*p*=0.951). The total UPDRS-ON score was significantly lower in the good-response group with a total score of 34.5 (*p*=0.025), and dyskinesia was more frequent in the good-response group (UPDRS-IV, *p*=0.023) (Table 3). Patients were divided in tremor dominant (TD) and postural instability and gait difficulty (PIGD) subtype based on UPDRS scores in accordance with Jancovic 1990 [11]. Only four patients were of TD subtype, and three (75%) of them had an effect >30%. Twenty-eight patients were of PIGD subtype, of which nine (32%) had an effect >30%.

The patients with PD had a significantly lower MoCA score where the median score was 27 compared to CS who had 29 as a median score (*p*=0.005). The patient with PD had a higher BDI score (*p* < 0.001) compared to CS (Table 1). There were no significant differences in these test results between patients with L-dopa response over or under 30%.

## 4. Discussion

In this study, the requirement of 30% improvement of motor symptoms with levodopa as a prerequisite in the current diagnostic criteria for PD was explored. We performed an evaluation of the levodopa response based on an acute effect of L-dopa in a group of patients with probable PD with both a self-reported good and poor medicine effect. While less than half of the patients had an effect >30%, the average improvement of motor symptoms using LCDT was 27.3%. We found no significant differences between the patients with improvement in UPDRS over or under 30%, with respect to demographic data, MoCA, BDI, and there were no indications of a relation to clinical subtypes being defined as either tremor dominant or PIGD. Our results showed that for many patients, a percentage improvement of motor UPDRS close to 30% was found using LCDT (Figure 1) [12, 13]. This lends support to the limit proposed in the MDS diagnostic criteria, but it also shows that the limit should be used with caution, and care should be taken to consider both acute and long-term effects and to interpret an UPDRS-III improvement below 30% cautiously including the possibility for potential fluctuations of response. We found a mean effect of LCDT <30%. As we specifically wanted to include participants who also reported a lack of effect, this does not reflect a general effect of L-dopa in the population with PD in the outpatient clinic. We evaluated 13 patients with a self-reported poor response and 24 patients with a self-reported good response; however, we found a marked discrepancy between patients' self-reported effect and the actual measured effect. Mostly, the patients tended to overestimate the effect of medication. This discrepancy may partly be based on bias in the use of only an acute evaluation [14].

The levodopa dose in the LCDT was set at a fixed level, but the patients' usual LEDD was variable. This could potentially have influenced the result, with an individual effect related to both usual medication and the level of disease progression. However, neither disease duration nor LEDD differed between the groups. In the diagnostic criteria, it is recommended to try a dose of levodopa of more than 600 mg in order to evaluate the effect properly. In our study, not all patients had a usual daily dose of levodopa of more than 600 mg, as proposed in the clinical diagnostic criteria; 12 patients had a dose of less than 600 mg LEDD. This may potentially have influenced the evaluation of the clinical effect; however, only four of the 13 patients estimating a poor medicine effect had a dose less than 600 mg/d. The level of usual medication may, however, influence the defined OFF situation, as a long-term effect of levodopa may be present in addition to the acute response. In this way, the patients with a higher usual dose may have a slightly higher (worse) UPDRS OFF compared to the patients with very low doses. This is the case both early and late in the disease [15]. We tried to minimize this by using a rigid medicine pause, but a long-term levodopa effect could still be present. This did however not seem to influence our results, as we found no correlation to LEDD.

The use of UPDRS to evaluate relative changes in symptoms has been discussed, and the new MDS-UPDRS was developed to improve evaluation of changes. But limitations with regard to precision of motor changes have still been described [16]. Previous validations have primarily looked at drug-naïve de-novo patients (deNoPa, PPMI) [5]. A 30% change in early disease stages may obviously not be directly comparable to a 30% change in later stages; however, no significant difference between the groups regarding disease duration or H&Y was seen in our study. A minimal relevant change is important [4], but this lends support to the notion that 30% should be regarded with some reservation.

A group of the patients who showed a good response in LDCT had reported a general poor response to medication. The expectation of a poor response could be based on unrealistic expectations by the patients in terms of effect. One example was a patient with tremor dominant disease and good levodopa response on LCDT. Because the tremor still sometimes occurred in a violent form, he experienced an unsatisfactory L-dopa effect. A difference in expected and measured response was evaluated in a study by Zolfaghari et al. where they compared self-reported Activities of Daily Living (MDS-UPDRS-II) with the objective clinical examination by MDS-UPDRS-III. They found that patients who had a high subjective score also reported more nonmotor symptoms, i.e., more nonlevodopa responsive symptoms [17]. We did not see any relation between UPDRS II and III in this way. Although the patients in our study who reported a poor medicine effect had a higher UPDRS-II score, it was not significantly different from the patients who reported a good response.

Patients with PD had a significantly lower MoCA score compared to the CS. This was expected, as cognitive impairment is more common in patients with PD than in the healthy population [18]. 27% of the patients had a score below cut-off 26, which indicate possible mild cognitive impairment (MCI) [9]. In a Swedish normative data study, the mean MoCA score was 26 in a population with an age range from 65 to 85 [19], which is lower than the median score we found in our CS and PD patients. However, we did not find any relation to the effect of medication.

The patients in our study had more depressive symptoms than CS according to the BDI questionnaire. In PD, depression is common and can occur at all stages of PD [20]. However, none of the included patients had a diagnosis of clinical depression, and the BDI scores did not exceed the limit for a depression. A study found that depressive symptoms had a negative impact on MoCA performance especially on executive function and attention in CS [21] and cognitive domains commonly affected in PD [22]. Therefore, signs of depression should probably be considered for the interpretation of the MoCA score. According to the MDS guidelines for diagnosis of MCI, patients with severe depression should be retested when their symptoms are improved or resolved [23]. As the patients in our study were tested after a night without medication, this may have had a negative effect on the concentration/performance in MoCA and on the BDI score.

### 4.1. Limitations

While this study highlights some important challenges in using a set cut-off regarding the L-dopa effect, it has limitations. The first nine patients UPDRS-III performance were not evaluated blinded.

The number of patients was limited, which means that it is not representative of all presentations of PD. However, as an evaluation of the use of a cut-off of 30% as a diagnostic test securing a correct diagnosis of PD, it is still illustrative in a group of patients with a diagnosis of PD. This group included patients with a disease duration of between 3 and 21 years, and all had a diagnosis of PD made by a movement disorder specialist.

The study design did not allow us to take different uptake of medication into account. It could be argued that a measured poorer effect was due to a reduced uptake. However, this also illustrates real life experience using the medicine effect as a marker for diagnosis.

While LCDT as an objective measure of L-dopa response has been widely accepted, both an acute and a more protracted effect of levodopa has been described [14]. A more appropriate evaluation of the effect might have been a stepwise increase of the dose of L-dopa, to see if patients with an acute response <30% eventually would reach the threshold as suggested by Albanese et al. [12].

## 5. Conclusion

We have performed a clinically relevant evaluation of levodopa response in a group of patients with PD with both a self-reported good and poor L-dopa response. An acute LDCT was performed as an objective measure, but a surprisingly large discrepancy was found between the subjectively reported usual response and the acute effect. As patients all otherwise had a diagnosis of PD, a good effect should be found for both the subjective and objective evaluation in accordance with diagnostic criteria. Our study sheds light on the importance of questioning the reported effect when making a diagnosis. The mean LCDT was close to 30% in most cases, but our study indicates that an acute effect of less than 30% should sometimes be expected.

In conclusion, this study showed that the LCDT can be used as a diagnostic support and that it is a valuable tool in addition to the self-reported effect from the patient. But also, that conclusions based on an acute effect of less than 30% should be evaluated with caution, as this was the case in 56.8% of the patients with PD in our study.

Care should be taken when interpreting UPDRS-III improvement below the specified cut-off value of 30% including considerations of potential fluctuations of response. Thus, the levodopa effect as a diagnostic tool should be evaluated with a nuanced view taking both the broader clinical picture and the current context into account. Also, the self-reported effect must be evaluated thoroughly.

## Figures and Tables

**Table 1:** Demographic data and MoCA score for patients with PD and control subjects.

Variables	PD (*n* = 37)	CS (*n* = 29)	*p* value
Age (y)	66 [47, 87]	63 [49, 80]	0.522
No. of men/women (%)	22/15 (59.5/40.5)	13/16 (44.8/55.2)	0.321
Education (y)	16 [7, 24]	15.5 [10, 20]	0.176
MoCA score	27 [20, 30]	29 [24, 30]	0.005
BDI	8 [0, 19]	1 [1, 12]	<0.001

PD, Parkinson's disease; CS, control subjects; MoCA, Montreal Cognitive Assessment; BDI, Beck's Depression Inventory. All values are reported as the median [minimum, maximum] or absolute frequency (relative frequency in %).

**Table 2:** Demographic data for PD with LDCT over and under 30% improvement.

Variable	PD LDCT ≤ 30% (*n* = 21)	PD LDCT > 30% (*n* = 16)	*p* value
Age (y)	65 [47, 78]	67 [49, 87]	0.591
No. of men/women (%)	12/9 (57.1, 42.9)	10/6 (62.5, 37.5)	1.000
Age at symptom onset (y)	57 [39, 71]	57.5 [39, 84]	0.771
Age at time of diagnosis (y)	61 [44, 73]	60.5 [39, 84]	0.976
Disease duration after onset of symptoms (y)	7 [4, 21]	11 [3, 16]	0.528
Disease duration after diagnosis (y)	5 [2, 12]	7.5 [3, 13]	0.076
Education (y)	17 [7, 24]	15 [9.5, 20]	0.042
MoCA score	27 [24, 30]	27 [20, 30]	0.596

PD LDCT ≤30%, PD acute levodopa challenge test with a change less than or equal to 30% improvement of UPDRS III; PD LDCT >30%, PD acute levodopa challenge test with more than 30% improvement of UPDRS III; MoCA, Montreal Cognitive Assessment. If participants had ≤12 years of education one point were added to the total MoCA score if < 30 to correct for education effects as recommended by the original validation study [9]. Three PD and three controls had ≤12 years of education and all three PD had 1 point added to their total score but only one control due to a score below 30. The median scores of MoCA for PD groups divided after the measured effect were both 27 (*p* 0.596). All values are reported as the median (minimum, maximum) or absolute frequency (relative frequency in %).

**Table 3:** Motor function and test results for patients with PD divided after the measured medicine effect.

Variable	PD LDCT ≤ 30% (*n* = 21)	PD LDCT > 30% (*n* = 16)	*p* value
Hand dominance, right/left (%)	17/4 (81, 19)	13/3 (81.2, 18.8)	1.000
Motor asymmetry, yes/no	16/5 (76.2, 23.8)	14/2 (87.5, 12.5)	0.675
Tremor as first symptom, yes/no	7/14 (33.3, 66.7)	9/7 (56.2, 43.8)	0.196
UPDRS total on	42 [15, 72]	34.5 [17, 53]	0.025
UPDRS-I	3 [0, 7]	2 [1, 6]	0.913
UPDRS-II	12 [3, 20]	10.5 [2, 18]	0.380
UPDRS-III motor score			
Off score	30 [10, 59]	34 [19, 49]	0.634
On score	26 [8, 57]	16.5 [6, 29]	0.006
Motor improvement in %	17.0 [−20.0, 27.6]	42.9 [31.0, 83.8]	<0.001
UPDRS-IV	2 [0, 9]	4 [0, 9]	0.023
Total LEDD (mg/day)	550 [140, 1140]	612 [164, 1460]	0.951
Modified Hoehn and Yahr stage	2 [1, 3]	2 [2, 3]	0.973
ADL	80 [60, 90]	90 [80, 90]	0.211
BDI	8 [0, 19]	8.5 [0, 16]	1.000

PD LDCT ≤ 30%, PD acute levodopa challenge test with a change less than or equal to 30% improvement of UPDRS III; PD LDCT > 30%, PD acute levodopa challenge test with more than 30% improvement of UPDRS III; UPDRS, Unified Parkinson's Disease Rating Scale; LEDD, Levodopa Equivalent Daily Dose; ADL, Schwab and England Activities of Daily Living Scale; BDI, Beck's Depression Inventory. The total daily L-dopa equivalent dose (LEDD) for each patient is calculated by using the conversion factors form Tomlinson [10]. We did not distinguish between L-dopa medicines with immediate or controlled release. All values are reported as the median (minimum, maximum) or absolute frequency (relative frequency in %).

## Data Availability

The data supporting the findings of this study are available on request from the corresponding author.
